# Predictors of futile recanalization after endovascular treatment in acute ischemic stroke: a multi-center study

**DOI:** 10.3389/fnins.2023.1279366

**Published:** 2023-11-28

**Authors:** Yu Sun, Eric Jou, Thanh N. Nguyen, Mohammad Mofatteh, Qingjia Liang, Mohamad Abdalkader, Zile Yan, Mingzhu Feng, Xinyuan Li, Guilan Li, Lanzhu Luo, Yuzheng Lai, Shuiquan Yang, Sijie Zhou, Zhiming Xu, Xiaodong Cai, Yimin Chen

**Affiliations:** ^1^Department of Neurology and Advanced National Stroke Center, Foshan Sanshui District People's Hospital, Foshan, China; ^2^Kellogg College, University of Oxford, Oxford, United Kingdom; ^3^Department of Neurology, Radiology, Boston University Chobanian & Avedisian School of Medicine, Boston, MA, United States; ^4^School of Medicine, Dentistry and Biomedical Sciences, Queen’s University Belfast, Belfast, United Kingdom; ^5^Department of Internal Medicine, Foshan Sanshui District People's Hospital, Foshan, China; ^6^Department of Radiology, Boston University Chobanian & Avedsian School of Medicine, Boston, MA, United States; ^7^The Second School of Clinical Medicine, Southern Medical University, Guangzhou, China; ^8^Medical Intern, Foshan Sanshui District People's Hospital, Foshan, China; ^9^Department of Neurology, Guangdong Provincial Hospital of Integrated Traditional Chinese and Western Medicine (Nanhai District Hospital of Traditional Chinese Medicine of Foshan City), Foshan, China; ^10^Department of Surgery of Cerebrovascular Diseases, First People's Hospital of Foshan, Foshan, China; ^11^Advanced Stroke Center Management Committee, Foshan Sanshui District People's Hospital, Foshan, China; ^12^Dean Office, Foshan Sanshui District People's Hospital, Foshan, China; ^13^Department of NeurologyThe Sixth Affiliated Hospital of Sun Yat-Sen University, Guangzhou, China; ^14^Biomedical Innovation Center, The Sixth Affiliated Hospital, Sun Yat-sen University, Guangzhou, China; ^15^Neuro International Collaboration (NIC), Foshan, China

**Keywords:** recanalization, endovascular thrombectomy, stroke, NIHSS, prognosis, futile recanalization, patient outcome

## Abstract

**Background and objectives:**

Endovascular thrombectomy (EVT) improves long-term outcomes and decreases mortality in ischemic stroke patients. However, a significant proportion of patients do not benefit from EVT recanalization, a phenomenon known as futile recanalization or reperfusion without functional independence (RFI). In this study, we aim to identify the major stroke risk factors and patient characteristics associated with RFI.

**Methods:**

This is a retrospective cohort study of 297 consecutive patients with ischemic stroke who received EVT at three academic stroke centers in China from March 2019 to March 2022. Patient age, sex, modified Rankin Scale (mRS), National Institute of Health Stroke Scale (NIHSS), Alberta stroke program early CT score (ASPECTS), time to treatment, risk factors and comorbidities associated with cerebrovascular diseases were collected, and potential associations with futile recanalization were assessed. RFI was successful reperfusion defined as modified thrombolysis in cerebral infarction (mTICI) ≥ 2b without functional independence at 90 days (mRS ≥ 3).

**Results:**

Of the 297 initial patients assessed, 231 were included in the final analyses after the application of the inclusion and exclusion criteria. Patients were divided by those who had RFI (*n* = 124) versus no RFI (*n* = 107). Older age (OR 1.041, 95% CI 1.004 to 1.073; *p* = 0.010), chronic kidney disease (OR 4.399, 0.904–21.412; *p* = 0.067), and higher 24-h NIHSS (OR 1.284, 1.201–1.373; *p* < 0.001) were independent predictors of RFI. Conversely, an mTICI score of 3 was associated with a reduced likelihood of RFI (OR 0.402, 0.178–0.909; *p* = 0.029).

**Conclusion:**

In conclusion, increased age, higher 24-h NIHSS and lack of an mTICI score of 3 were independently associated with RFI and have potential prognostic values in predicting patients that are less likely to respond to EVT recanalization therapy.

## 1 Introduction

Endovascular thrombectomy (EVT) has been demonstrated to improve outcomes in select patients with large vessel occlusion ischemic stroke up to the 24-h time window, with significant benefits including a reduction in long-term functional disability and mortality (Hong et al., 2015; Goyal et al., 2016; Lansberg et al., 2019; Campbell and Nguyen, 2022). However, approximately half of the patients do not achieve functional independence despite successful reperfusion, a phenomenon that has otherwise been termed futile recanalization (Pan et al., 2021; Shahid et al., 2022). Recent studies have shifted away from utilizing the term futile reperfusion or futile recanalization because some of these patients can still have a quality of life, and the endovascular intervention hence was not futile (Seker et al., 2022). A patient who achieves a modified Rankin Scale (mRS) of 3 may depend on others for daily activities and remains independent for ambulation. As this may still be considered a meaningful outcome for patients, the term reperfusion without functional independence (RFI) may be a preferred term to describe this phenomenon (Seker et al., 2022; Weyland et al., 2022).

Predicting functional outcomes of acute stroke patients can use early neurological improvements as a surrogate marker (Chen et al., 2023). One of the major challenges is the identification of variables that can predict early neurological improvements after EVT, posing a challenge to predict longer-term outcomes in patients undergoing EVT requires (Lai et al., 2023). We previously demonstrated that multiple factors, such as diabetes mellitus history, pre-stroke mRS, longer last known well-to-puncture time, and the number of mechanical thrombectomy passes are the predictors of failure of early neurological improvement (Lai et al., 2023). Furthermore, other issues, such as challenging arterial anatomy, may prevent successful and timely EVT, which can be resolved by crossover from femoral to radial access (Chen et al., 2023).

Importantly, EVT and recanalization are not without risk to patients. Potential drawbacks include extracranial and intracranial complications such as vessel injury (e.g., dissection), emboli to new territory, and intracranial hemorrhage, respectively (Darkhabani et al., 2012; Pilgram-Pastor et al., 2021). Identifying predictors of RFI is important to provide prognostic information to patients and their families with incoming large vessel occlusion (LVO), and to help them arrive at an informed decision to undergo EVT or of expectations post-EVT. In this study, we aim to identify clinical parameters, including risk factors and patient characteristics that are associated with RFI.

## 2 Materials and methods

### 2.1 Patients

This was a retrospective analysis of prospectively collected data from consecutive ischemic stroke patients who underwent endovascular therapy from March 2019 to March 2022 at three academic comprehensive stroke centers in China. The data were derived from the Big Data Observatory Platform for stroke in China and from the hospital data platform.

The inclusion criteria were age ≥ 18 years old as the study focused on the adult population, patients presenting within 24 h from time last seen well, pre-EVT Alberta stroke program early CT score (ASPECTS) 6 or greater, occlusion of the ICA, M1, M2 or basilar artery, premorbid mRS < 2, and final reperfusion of modified thrombolysis in cerebral infarction (mTICI) > 2b/3.

**Figure 1:**
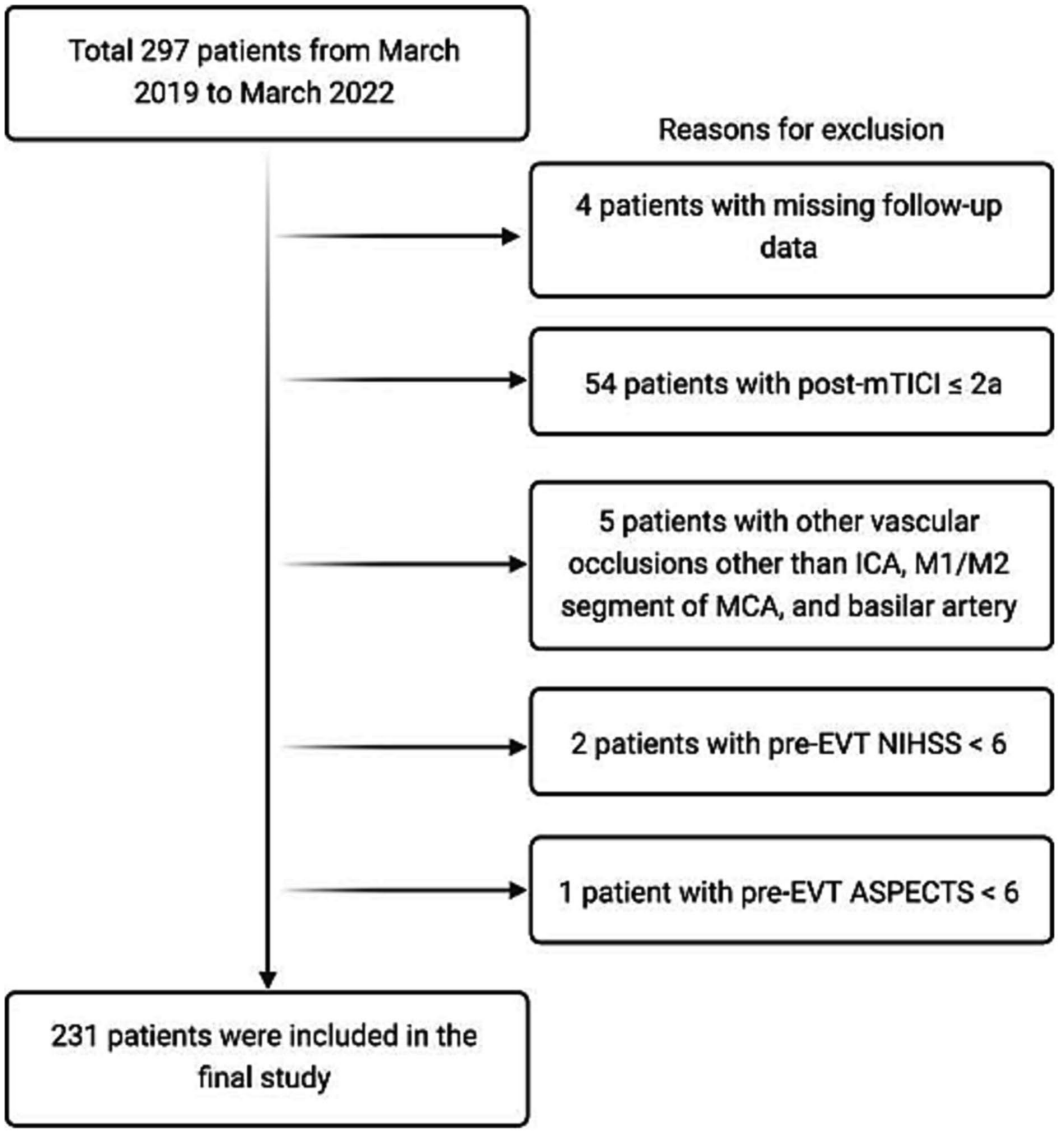
An overview of patient inclusion in the current study. EVT, endovascular thrombectomy; mTICI, modified thrombolysis in cerebral infarction; NIHSS, National Institute of Health Stroke Scale.

Exclusion criteria were missing data at follow-up, post-mTICI ≤2a, other vascular occlusion not listed above, pre-EVT NIHSS < 6, pre-EVT ASPECTS < 6, premorbid mRS ≥ 3, over 24 h from onset. An overview of patient inclusion is demonstrated in Figure 1.

The study protocol was approved by the hospital’s institutional review board. All procedures performed in the study involving human participants were in accordance with the ethical standards of the institutional and/or national research committee and with the 1964 Declaration of Helsinki and its later amendments or comparable ethical standards.

### 2.2 Data collection

We collected the following data and assessed potential associations with futile recanalization: age, sex, risk factors of cerebrovascular disease, premorbid modified Rankin Scale (mRS), door-to-needle time (DNT), onset-to-needle time (ONT), door–to-puncture time (DPT), last-known-normal-to-puncture time (LKNPT), door-to-recanalization time (DRT), modified thrombolysis in cerebral Infarction (mTICI) post thrombectomy. Patient outcomes were evaluated by 3-month mRS after EVT. Functional independence was considered as mRS of 0–2 at 3 months. Walking independence was defined as mRS of 3. An unfavorable outcome was defined as an mRS of 3–6 at 3 months.

Successful reperfusion was defined as mTICI ≥2b (Linfante et al., 2016), while RFI was defined as successful recanalization (mTICI ≥2b after thrombectomy) without functional dependence (mRS ≥ 3 at 90 days) following EVT (Zang et al., 2020; Seker et al., 2022).

Neurologists measured and recorded the National Institute of Health Stroke Scale (NIHSS) at admission and entered the data into the platform prospectively. Post-EVT NIHSS was performed by the interventionist or neurologist. The patients were followed up by trained stroke nurses or neurologists by telephone or in outpatient. Because this is a national stroke center project, we are required to follow up on the 3-month mRS of all EVT patients.

### 2.3 Statistical analysis

The non-parametric Mann–Whitney U test was performed by using IBM SPSS version 27 (IBM-Armonk, NY) to analyze non-normally distributed continuous data, reported as medians, along with the interquartile range (IQR). Normally distributed data are reported as means with corresponding standard deviations (SD) and compared using the Student’s t-test. Results were considered statistically significant if the *p*-value was less than 0.05. The independent factors were further analyzed by backward selection multivariate binary regression.

## 3 Results

A total of 297 patients were initially assessed and evaluated for eligibility. Four patients were excluded due to lack of follow-up data, 54 were excluded for post-mTICI ≤2a, 5 patients had other vascular occlusions not eligible for this study, and another 3 were excluded due to having a pre-EVT NIHSS or ASPECTS outside the inclusion criteria, leaving a final 231 patients included in this study.

Of the 231 patients analyzed, all of whom achieved successful recanalization via EVT, 107 were deemed to have recanalization with functional independence (mRS < 2), while 124 had an mRS of ≥3 at 90 days after EVT. Comparison of baseline characteristics revealed that there was no difference in sex across the EVT or MM groups (70.97 and 67.29%, respectively, *p =* 0.55) while increasing age was associated with RFI (*p <* 0.001), with a mean age of 66.87 ± 12.17 years and 61.10 ± 12.75 years in the RFI and meaningful groups, respectively (Table 1).

**Table 1:** Baseline characteristics of patients with successful recanalization (mTICI 2b-3).

	RFI	Meaningful Recanalization	X^2^/t/z	*p*
Number	124	107		
Age Mean ± SD	66.87 ± 12.17	61.10 ± 12.75	−3.513	0.001*
Male, *n*, %	88 (70.97%)	72 (67.29%)	0.365	0.546
Risk factors				
Hypertension, *n*, %	76 (61.29%)	57 (53.27%)	1.512	0.219
Diabetes mellitus, n, %	34 (27.42%)	14 (13.08%)	7.170	0.007*
CAD, n, %	27 (21.77%)	10 (9.35%)	6.595	0.010*
Atrial fibrillation, *n*, %	42 (33.87%)	35 (32.71%)	0.035	0.852
Prior Stroke, *n*, %	28 (22.58%)	18 (16.82%)	1.194	0.274
Hyperlipemia, *n*, %	20 (16.13%)	22 (20.56%)	0.758	0.384
Chronic kidney disease, *n*, %	14 (11.29%)	4 (3.74%)	4.559	0.033*
Current Smoker, *n*, %	19 (15.32%)	30 (28.04%)	5.556	0.018*
NIHSS Pre-EVT (IQR)	16.00 (14.00, 21.00)	13.00 (10.00, 17.00)	−4.300	0.000*
ASPECTS pre-treatment (IQR)	8.00 (8.00, 9.00)	9.00 (8.00, 9.00)	−0.821	0.412
mRS pre-treatment (IQR)	0.00 (0.00, 0.00)	0.00 (0.00, 0.00)	−1.141	0.254
Occlusion vessels				
ICA *n*, %	27 (21.77%)	16 (14.95%)	1.764	0.184
MCA-M1 *n*, %	40 (32.26%)	57 (53.27%)	10.412	0.001*
MCA-M2 *n*, %	9 (7.26%)	6 (5.61%)	0.258	0.612
Tandem *n*, %	20 (16.13%)	16 (14.95%)	0.060	0.806
Basilar *n*, %	28 (22.58%)	12 (11.21%)	5.182	0.023*
Toast type				
Large artery atherosclerosis	57 (45.97%)	56 (52.34%)	1.524	0.677
Cardioembolic	61 (49.19%)	45 (42.06%)		
Stroke of other determined etiology	2 (1.61%)	3 (2.80%)		
Stroke of undetermined etiology	4 (3.23%)	3 (2.80%)		
IV thrombolysis, *n*, %	53 (42.74%)	43 (40.19%)	0.154	0.694
DNT	45.00 (32.00，60.00)	42.50 (30.00, 53.00)	−0.892	0.372
ONT	139.00 (100.00, 179.00)	121.00 (95.00, 178.25)	−0.754	0.451
DPT (IQR), min	152.00 (117.25, 205.00)	128.00 (105.00, 190.00)	−2.190	0.029*
DRT (IQR), min	242.00 (183.25, 314.75)	207.00 (155.00, 259.00)	−2.641	0.008*
PRT (IQR), min	65.00 (44.25, 106.00)	55.00 (35.00, 85.00)	−2.187	0.029*
LKNPT (IQR), min	297.50 (207.75, 434.50)	290.00 (195.00, 470.00)	−0.651	0.515
mTICI 3	48 (38.71%)	66 (61.68%)	12.127	< 0.001*
No of EVT attempts	1.00 (1.00, 2.00)	1.00 (1.00, 2.00)	−0.764	0.445
sICH, *n*, %	19 (15.32%)	0 (0.00%)	17.865	< 0.001*
24 h NIHSS	17.00 (12.00, 25.00)	5.00 (2.00, 11.00)	−10.069	< 0.001*

*Denotes significance. CAD, coronary artery disease; CKD, chronic kidney disease; DPT, door-to-puncture time; DRT, door-to-recanalization time; EVT, endovascular thrombectomy; IV, intravenous; LKNPT, last-known normal-to-puncture time; mTICI, modified thrombolysis in cerebral infarction; NIHSS, National Institute of Health Stroke Scale.

Potential associations of major risk factors of stroke and patient comorbidities to outcome after recanalization were also assessed (Table 1). There was no difference in the proportion of patients with hypertension between the RFI and meaningful recanalization groups (61.29 and 53.27%, respectively, *p* = 0.219), and similarly for other cardiovascular risk factors including atrial fibrillation (33.87% vs. 32.71%, *p* = 0.852) and hyperlipidemia (16.13% vs. 20.56%, *p* = 0.384). Importantly, prior stroke events were not associated with poor outcomes after EVT (*p* = 0.274), whilst symptomatic intracerebral hemorrhage (sICH) was associated with an increased risk of RFI (*p* < 0.0001).

Initial analyses revealed higher rates of diabetes mellitus (DM; 27.42% vs. 13.08%, *p* = 0.007), coronary artery disease (CAD; 21.77% vs. 9.35%, *p* = 0.010) and chronic kidney disease (CKD; 11.29% vs. 3.74%, *p* = 0.033) in the RFI group compared to those with meaningful recanalization, respectively (Table 1). Of these, further analyses through binary regression demonstrated that DM (*p = 0.039*) independently predicts RFI (Table 2). Similar to DM, older age was also independently associated with higher rates of RFI (*p =* 0.010). Intriguingly, whilst initial analysis revealed that current smokers may have reduced risk of RFI (*p =* 0.018), this association was not independent upon further scrutiny (Tables 1, 2). Furthermore, whilst occlusion of the basilar artery was enriched in the futile recanalization group compared to those with meaningful recanalization (22.58% vs. 11.21%, *p* = 0.023), this association was not independent after regression.

**Table 2:** Independent predictors of RFI by binary regression.

	OR	95% CI	*p*
Age	1.041	1.010 ~ 1.073	0.010
DM	2.829	1.052 ~ 7.608	0.039
CKD	4.399	0.904 ~ 21.412	0.067
DRT	1.004	1.000 ~ 1.009	0.079
NIHSS 24 Hour	1.284	1.201 ~ 1.373	0.000
mTICI 3	0.402	0.178 ~ 0.909	0.029

Model by logistics binary regression (backward selection): age, diabetes mellitus (DM), coronary artery disease (CAD), CKD (chronic kidney disease), smoker, M1 occlusion, BA occlusion, DPT, DRT, PRT, NIHSS 24 Hour, mTICI 3, no of EVT attempts, sICH.

**Figure 2:**
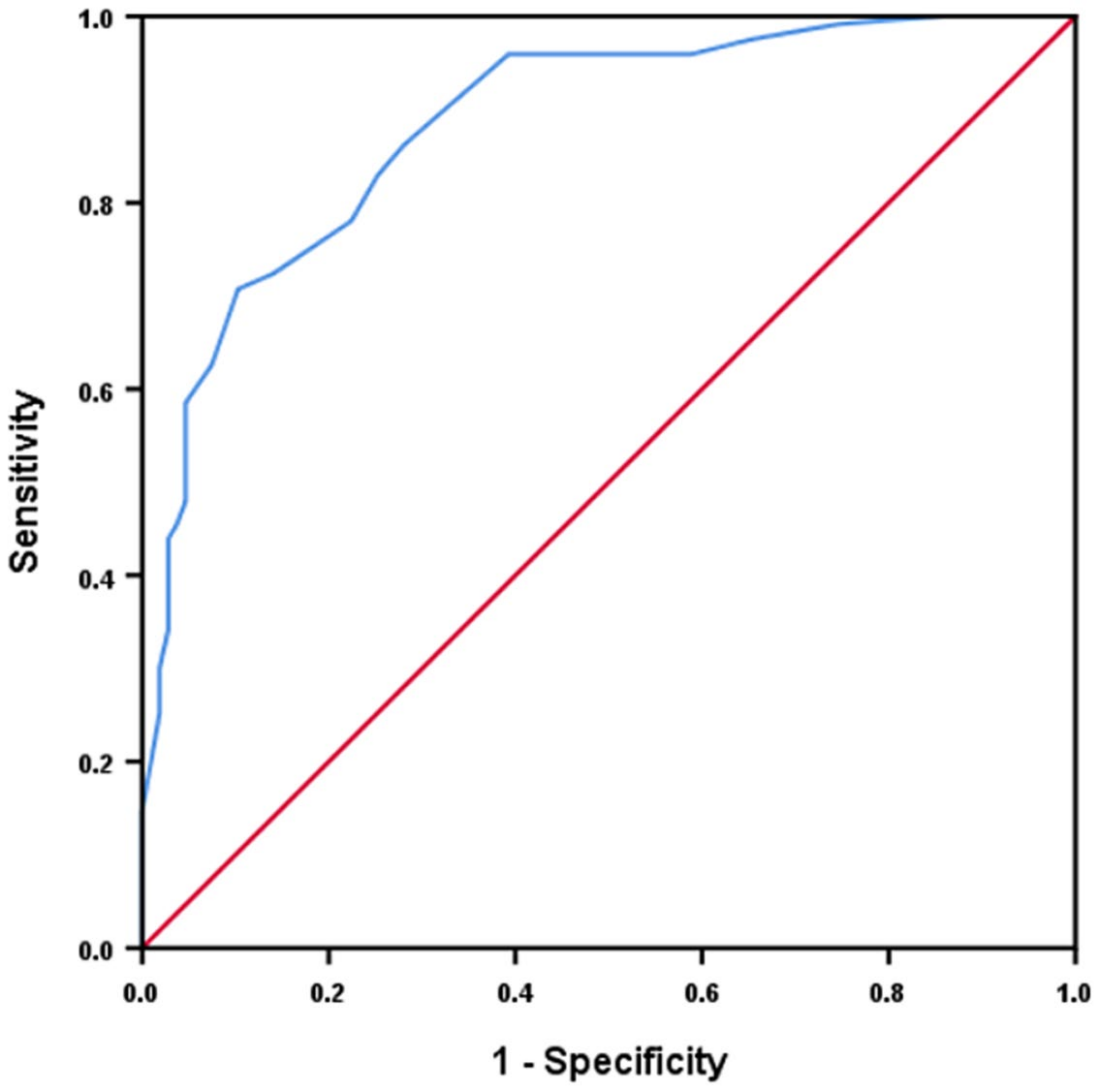
24-h NIHSS predicting reperfusion without functional independence.

In terms of scoring systems, pre-treatment NIHSS (*p* < 0.001), but not ASPECTS (*p* = 0.412) or mRS (*p = 0.254*), was associated with improved outcomes and reduced incidence of RFI (Table 1). On the other hand, a high 24-h NIHSS was found to predict RFI (Figure 2) (OR 1.041, 95% CI 1.004–1.073; *p =* 0.010) (Table 2).

**Figure 3:**
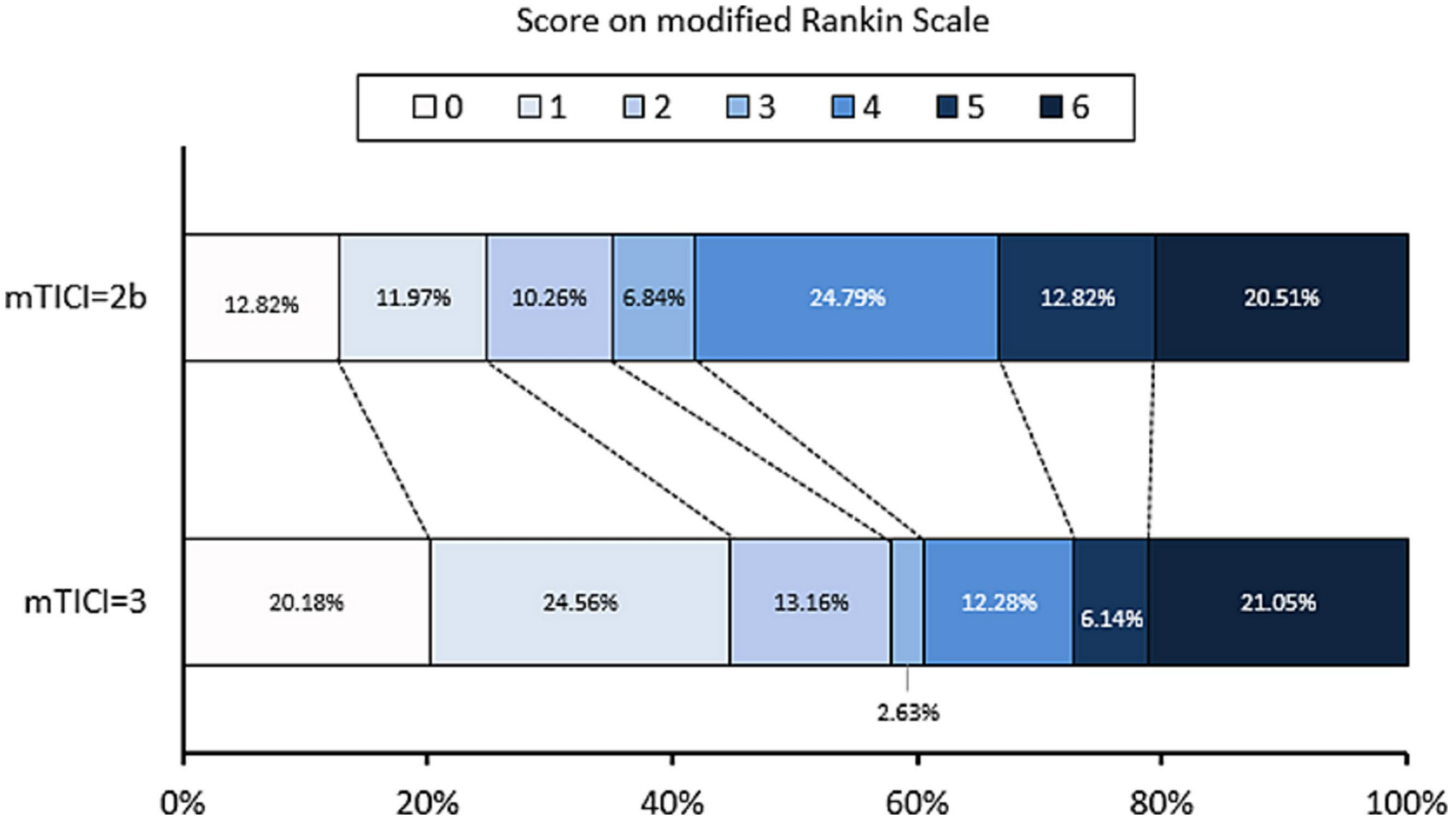
Distribution of 3-month mRS of mTICI = 2b and mTICI = 3.

**Figure 4:**
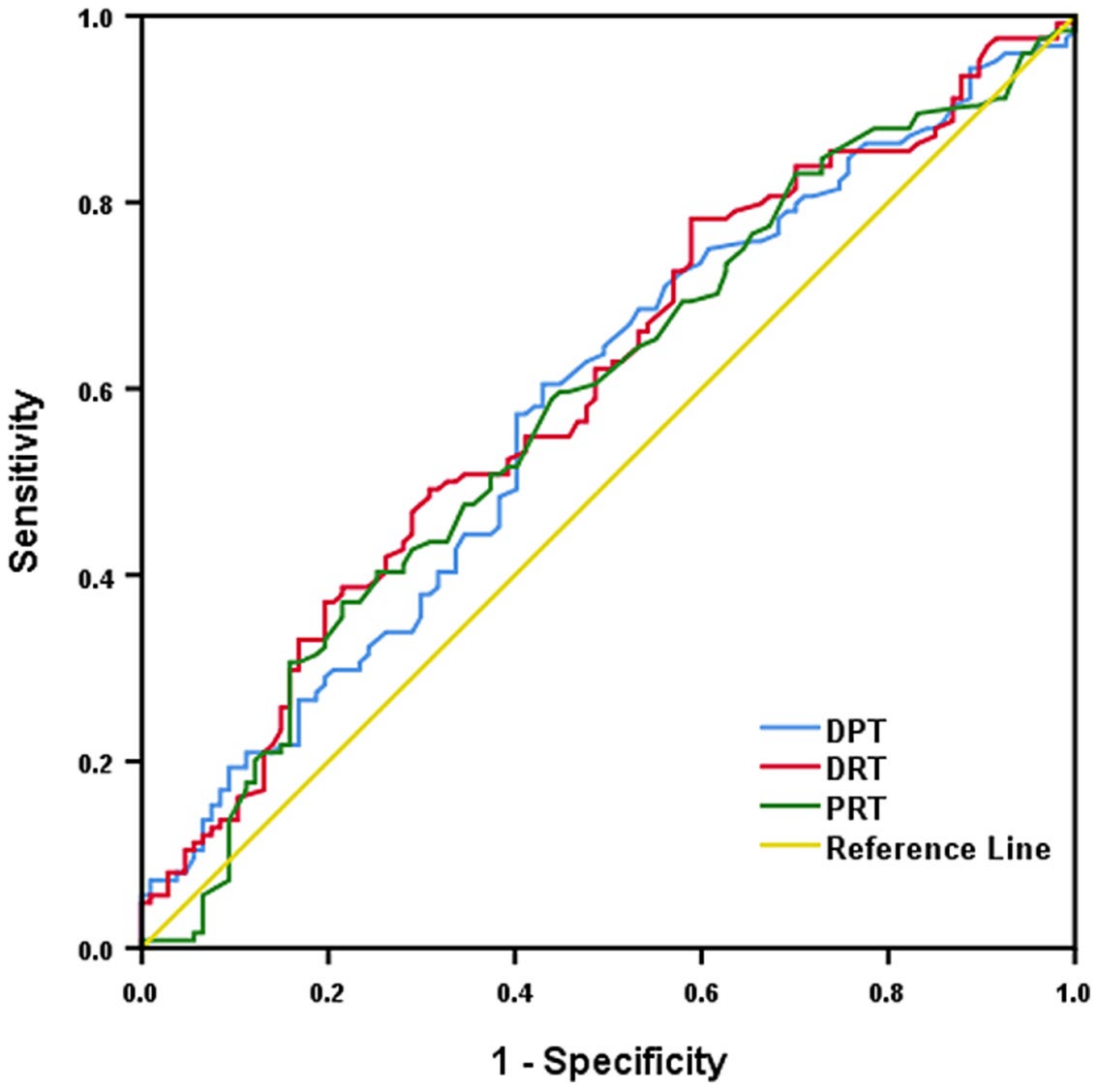
DPT, DRT, PRT predicting futile recanalization.

Furthermore, an mTICI score of 3 was independently associated (OR 0.402, 0.178–0.909; *p = 0.029*) with meaningful recanalization (Table 2; Supplementary Table S1; Figure 3), while the number of EVT attempts did not predict RFI. Prolonged DPT and DRT may also be associated with futile recanalization (Figure 4). DPT and DRT were higher in the RFI group compared to the meaningful recanalization (*p =* 0.029 and *p =* 0.008, respectively).

## 4 Discussion

Whilst EVT recanalization has been associated with significant improvements in long-term neurological outcomes and reduced mortality in patients with ischemic stroke in several RCTs, recent multi-center RCTs and meta-analyses indicated that in up to 60% of patients, such recanalization is “futile” with minimal improvement to functional outcome (Hussein et al., 2010; Gomis and Dávalos, 2014; Hong et al., 2015; Goyal et al., 2016; Nie et al., 2018). Understanding the factors that predict RFI is critical as this will allow maximal benefits to stroke patients that are likely to respond to therapy while allowing early application of alternative treatment regimens while minimizing iatrogenic harm to those where recanalization may not result in desired outcomes. In this study, we analyzed the association of major risk factors of stroke, temporal parameters to EVT treatment delivery, and patient characteristics and comorbidities to outcome after recanalization, and found increased age, DM, an mTICI score of <3, and a high 24-h NIHSS to be independently associated with RFI. We defined RFI as an mRS ≥ 3 at 90 days after EVT, which is consistent with most studies in the literature (Hussein et al., 2010; Nie et al., 2018). Patients with these aforementioned characteristics are less likely to benefit from EVT recanalization alone. Alternative adjunct therapies may be required and should be investigated in future studies, particularly in these patients, in order to achieve early neural protection with the potential to improve long-term outcomes.

A recent study found baseline brain atrophy to be associated with RFI, and this effect was independently amplified by increased patient age (Pedraza et al., 2020). Consistent with this, our present study and previous reports have similarly identified old age as an independent risk factor for RFI (Pan et al., 2021; Deng et al., 2022; Ni et al., 2022). Conversely, whilst one study found female sex and a higher NIHSS at admission to be associated with RFI (Ni et al., 2022), in the present study, we observed no predictive value of sex or pre-EVT NIHSS. As similar criteria for successful reperfusion and RFI were used in their study and ours, these discrepancies are likely due to differences in patient demographics, such as a higher average age in their cohort (72 in their study). Correlations between sex and RFI are often contradictory and inconsistent in the literature, leading to some authors attributing any associations of sex and RFI to coincidence (Deng et al., 2022). Alternatively, correlations between sex and recanalization outcome may be due to differences in hormones and coagulation (Reeves et al., 2008; Hussein et al., 2010). Although we found no association between pre-EVT NIHSS to RFI, our results demonstrate a correlation between the latter with increased 24-h NIHSS. Altogether, these findings indicate that early NIHSS can have long-term prognostic value in predicting RFI and outcome (Chen et al., 2022), and the optimal timing for measuring NIHSS may depend on patient characteristics such as age.

Interestingly, whilst our initial analyses identified CKD, CAD and DM to be associated with RFI, DM was independently associated with the latter upon further analyses, despite the strong association and many shared risk factors between CAD with stroke (Sobiczewski et al., 2013). Patients with CKD have an increased risk of stroke and an increased risk of stroke mortality by 30-fold (Chavda et al., 2021). The mechanisms behind the association of CKD and stroke are highly complex and likely involve bi-directional interplay between cerebral and renal pathways, shared vascular co-morbidities such as hypertension, and pathological changes to the vasculature associated with renal failure such as increased atherosclerosis secondary to malnutrition and inflammation (Chavda et al., 2021). Thrombolytic treatments for stroke have been associated with sICH development in CKD patients (Nayak-Rao and Shenoy, 2017). In this study, we also observed an association of sICH with RFI. Our findings, therefore, identify CKD to be an important comorbidity in predicting RFI compared to other diseases traditionally associated with stroke incidence such as DM and CAD. Future studies are required to improve our understanding of how CKD contributes to RFI.

Other potential factors that may affect the rate of RFI include the initial imaging modality used to select patients for EVT and biochemical markers, which we did not access in this study. A previous study indicated that CT-based selection for EVT was associated with increased RFI compared to MRI, despite similar rates of overall EVT after imaging and a 30-min delay to EVT in MRI-selected patients compared to those selected by CT (Meinel et al., 2020). However, we do not know about the patients who were excluded from EVT on the basis of MRI or CT selection who might have derived benefits. Another study showed that the imaging modality in the late 6–24 h window did not impact differences in patient outcomes or the development of RFI (Nguyen et al., 2022; Seker et al., 2022). With the expansion of EVT eligibility criteria now to include patients with large ischemic core infarctions with ASPECTS 3– 5, non-contrast CT will likely suffice for patient selection, and advanced imaging may not be as critical in-patient selection (Huo et al., 2023; Sarraj et al., 2023). Of note, some of these large ischemic core trials were chosen as primary outcome mRS 0–3, or independent ambulation, because this may be a meaningful outcome in patients who present with the greater ischemic territory of infarction (Yoshimura et al., 2022).

Also, we found associations between DPT or DRT and RFI (Figure 3); therefore, we should seize any opportunities to shorten the stroke treatment time and improve outcome (Chen et al., 2022; Yang et al., 2022). On the other hand, previous studies have found that an early increase in body temperature is associated with EVT outcome (Chen et al., 2022), and certain biomarkers that can be measured in patient blood samples, such as increased matrix metalloproteinase-9 (MMP-9) and tenascin-C, but not CRP, were associated with RFI (Zang et al., 2020). MMP-9 is part of a family of proteins with important roles in the degradation of the extracellular matrix, and serves as a marker for blood–brain barrier disruption, potentially explaining its association with worse outcomes and RFI (Turner and Sharp, 2016). Similarly, tenascin-C is found in the extracellular matrix and is associated with poor prognosis in stroke due to its neuroinflammatory properties (Zaidi et al., 2019; Okada and Suzuki, 2020), while CRP, a commonly used clinical marker for non-specific general inflammation, did not correlate with futile recanalization (Zang et al., 2020). Collectively, these findings indicate that clinical parameters, and patient blood biomarkers after stroke, may also have predictive value in addition to intrinsic patient characteristics and comorbidities, to identify patients susceptible to RFI.

This study has some limitations which need to be mentioned. This study was limited by the fact that it enrolled patients from three large stroke centers in China only. Therefore, the population lacked diversity compared to similar international studies. However, scarce information regarding RFI from low and middle-income countries can highlight disparities and differences in the treatment of stroke patients globally. Furthermore, the study was conducted retrospectively, which can introduce bias. The sample size was relatively small, which can further introduce some bias in our analysis. In addition, we did not include all possible prognostic variables, such as collateral status, dementia, etc. Despite these shortcomings, we believe data from the current study can facilitate future studies to understand the status of EVT and its outcome in developing nations. Future international prospective studies with larger sample size which investigate multiple prognostic variables are required to validate and expand these findings.

## 5 Conclusion

To conclude, in this study, we identified older age, mTICI of below 3, and higher 24 h NIHSS to be independent predictors of RFI. Understanding the factors that contribute to RFI will allow the development of prognostic tests to identify patients that may benefit from adjunctive pharmacological or neuroprotective therapies where EVT recanalization is likely to be associated with unfavorable outcomes.

## Data availability statement

The raw data supporting the conclusions of this article will be made available by the authors, without undue reservation.

## Ethics statement

The studies involving humans were approved by the Foshan Sanshui District People’ Hospital Board Review. The studies were conducted in accordance with the local legislation and institutional requirements. The ethics committee/institutional review board waived the requirement of written informed consent for participation from the participants or the participants’ legal guardians/next of kin because in accordance with national and regional laws and regulations.

## Author contributions

YS: Conceptualization, Data curation, Formal analysis, Investigation, Methodology, Visualization, Writing – review & editing. EJ: Conceptualization, Formal analysis, Writing – original draft. TN: Writing – review & editing. MM: Project administration, Writing – review & editing. QL: Data curation, Formal analysis, Investigation, Writing – review & editing. MA: Writing – review & editing. ZY: Data curation, Formal analysis, Writing – review & editing. MF: Data curation, Formal analysis, Investigation, Writing – review & editing. XL: Data curation, Formal analysis, Investigation, Writing – review & editing. GL: Data curation, Formal analysis, Investigation, Writing – review & editing. LL: Data curation, Formal analysis, Investigation, Writing – review & editing. YL: Data curation, Formal analysis, Investigation, Writing – review & editing. SY: Data curation, Formal analysis, Investigation, Validation, Writing – review & editing. SZ: Data curation, Formal analysis, Investigation, Validation, Writing – review & editing. ZX: Conceptualization, Data curation, Formal analysis, Funding acquisition, Investigation, Methodology, Resources, Supervision, Validation, Visualization, Writing – original draft. XC: Conceptualization, Data curation, Formal analysis, Funding acquisition, Investigation, Methodology, Resources, Supervision, Validation, Visualization, Writing – original draft. YC: Conceptualization, Data curation, Formal analysis, Funding acquisition, Investigation, Methodology, Resources, Supervision, Validation, Visualization, Writing – original draft, Writing – review & editing.
